# Detecting imbalanced expression of SNP alleles by minisequencing on microarrays

**DOI:** 10.1186/1472-6750-4-24

**Published:** 2004-10-22

**Authors:** Ulrika Liljedahl, Mona Fredriksson, Andreas Dahlgren, Ann-Christine Syvänen

**Affiliations:** 1 Molecular Medicine, Department of Medical Sciences, Uppsala University, Uppsala, Sweden

## Abstract

**Background:**

Each of the human genes or transcriptional units is likely to contain single nucleotide polymorphisms that may give rise to sequence variation between individuals and tissues on the level of RNA. Based on recent studies, differential expression of the two alleles of heterozygous coding single nucleotide polymorphisms (SNPs) may be frequent for human genes. Methods with high accuracy to be used in a high throughput setting are needed for systematic surveys of expressed sequence variation. In this study we evaluated two formats of multiplexed, microarray based minisequencing for quantitative detection of imbalanced expression of SNP alleles. We used a panel of ten SNPs located in five genes known to be expressed in two endothelial cell lines as our model system.

**Results:**

The accuracy and sensitivity of quantitative detection of allelic imbalance was assessed for each SNP by constructing regression lines using a dilution series of mixed samples from individuals of different genotype. Accurate quantification of SNP alleles by both assay formats was evidenced for by R^2 ^values > 0.95 for the majority of the regression lines. According to a two sample t-test, we were able to distinguish 1–9% of a minority SNP allele from a homozygous genotype, with larger variation between SNPs than between assay formats. Six of the SNPs, heterozygous in either of the two cell lines, were genotyped in RNA extracted from the endothelial cells. The coefficient of variation between the fluorescent signals from five parallel reactions was similar for cDNA and genomic DNA. The fluorescence signal intensity ratios measured in the cDNA samples were compared to those in genomic DNA to determine the relative expression levels of the two alleles of each SNP. Four of the six SNPs tested displayed a higher than 1.4-fold difference in allelic ratios between cDNA and genomic DNA. The results were verified by allele-specific oligonucleotide hybridisation and minisequencing in a microtiter plate format.

**Conclusions:**

We conclude that microarray based minisequencing is an accurate and accessible tool for multiplexed screening for imbalanced allelic expression in multiple samples and tissues in parallel.

## Background

Single nucleotide polymorphisms (SNPs) are highly abundant in the human genome, appearing on average at 0.1% of the nucleotide positions [1]. Thus, each gene or transcriptional unit will contain multiple SNPs that potentially give rise to sequence variation between individuals and tissues on the level of RNA. Recent studies indicate that differences in the expression levels of the alleles of heterozygous SNPs may occur frequently for human genes [2-6]. Imbalanced allelic expression was detected in foetal liver or kidney tissues for more than half of 602 genes analysed, and one third of the genes displayed more than four-fold differences in allelic expression [3]. Another study detected lower levels of allelic imbalance for one fifth of 129 genes analysed in lymphoblastoid cell lines [4].

Non-synonymous SNPs in coding regions of genes may be functional by altering an amino acid, which in turn may affect the structure and function of the encoded protein, while synonymous SNPs may have functional consequences by affecting the stability or folding of mRNA transcripts. Intronic SNPs may give rise to alternatively spliced mRNAs, while SNPs in 5'- or 3'-untranslated mRNA regions may affect the stability or processing of the RNA. Moreover, SNPs in non-protein coding regions of genes that affect binding of regulatory factors may cause imbalanced expression of SNP alleles. This form of genetic variation has been suggested as a common cause of both normal and disease-related inter-individual variation in complex phenotypes [7]. Clearly, methods with high accuracy that can be used in a high throughput setting are needed for systematic surveys of expressed sequence variation and its molecular causes.

Owing to the high sequence specificity of nucleotide incorporation by DNA-polymerases, single nucleotide primer extension has proven to allow quantitative determination of SNPs in genomic DNA in several studies and assay formats (for a review, see Syvänen 2001 [8]). A frequently used quantitative application of the method is to determine SNP allele frequencies in pooled DNA samples [9-13]. The rationale for detecting imbalanced expression of the two alleles of a heterozygous SNP by minisequencing is to measure the ratio between the amounts of labelled nucleotides incorporated in the minisequencing reactions for the two SNP alleles in RNA (cDNA) samples from the tissue of interest. These ratios are then compared to the corresponding ratio measured in genomic DNA, where the two alleles are present in an equimolar ratio [2,4,14-16]. Imbalanced expression of the alleles of a SNP is revealed by a difference in the ratios measured in the RNA and DNA samples.

We are currently using microarray based minisequencing for multiplex genotyping of SNPs. Our custom-made microarrays permit the genotyping of up to 100 SNPs in 80 samples per standard microscope slide, either using immobilised minisequencing primers [17,18] or using a "tag-array" format [19,20] of the method [13]. The purpose of this study was to evaluate the performance of these two microarray formats in quantitative determination of SNP alleles on the RNA level as alternatives with higher multiplexing capacity than previously used primer extension methods in which the SNPs are analysed in individual reactions. Using these systems, we were able to detect significant differences in the amounts of the two alleles of heterozygous SNPs on the RNA level.

## Results

We used a panel of ten coding SNPs in five genes to choose the optimal microarray based minisequencing strategy for multiplex, quantitative genotyping of SNPs in DNA and RNA samples. The selected SNPs were located in genes shown by reverse transcriptase PCR analysis to be expressed in one or both of two endothelial cells lines, HUVEC (human umbilical vein endothelial cells) and HAEC (human aortic endothelial cells) that served as our model cell lines in this study (data not shown). We evaluated two formats of microarray based minisequencing by performing five parallel assays with each method for each sample in the evaluation. The SNPs were analysed in both DNA polarities and the evaluation of the methods was based on the DNA polarity yielding the highest signal-to-noise ratio.

In Method I, immobilised minisequencing primers are extended with fluorescently labelled ddNTPs in reactions performed on the microarray surface after annealing of the multiplex PCR products to the primers [18,21]. In Method II, cyclic primer extension reactions are performed in solution in the presence of 5'-tagged minisequencing primers, PCR products and fluorescent ddNTPs [22,23]. After the cyclic reactions the extended primers are captured on a microarray surface carrying immobilised oligonucleotides complementary to the 5'-tag sequences on the minisequencing primers. Both these systems are performed in an "array-of arrays" format developed previously in our laboratory [24].

**Figure 1:**
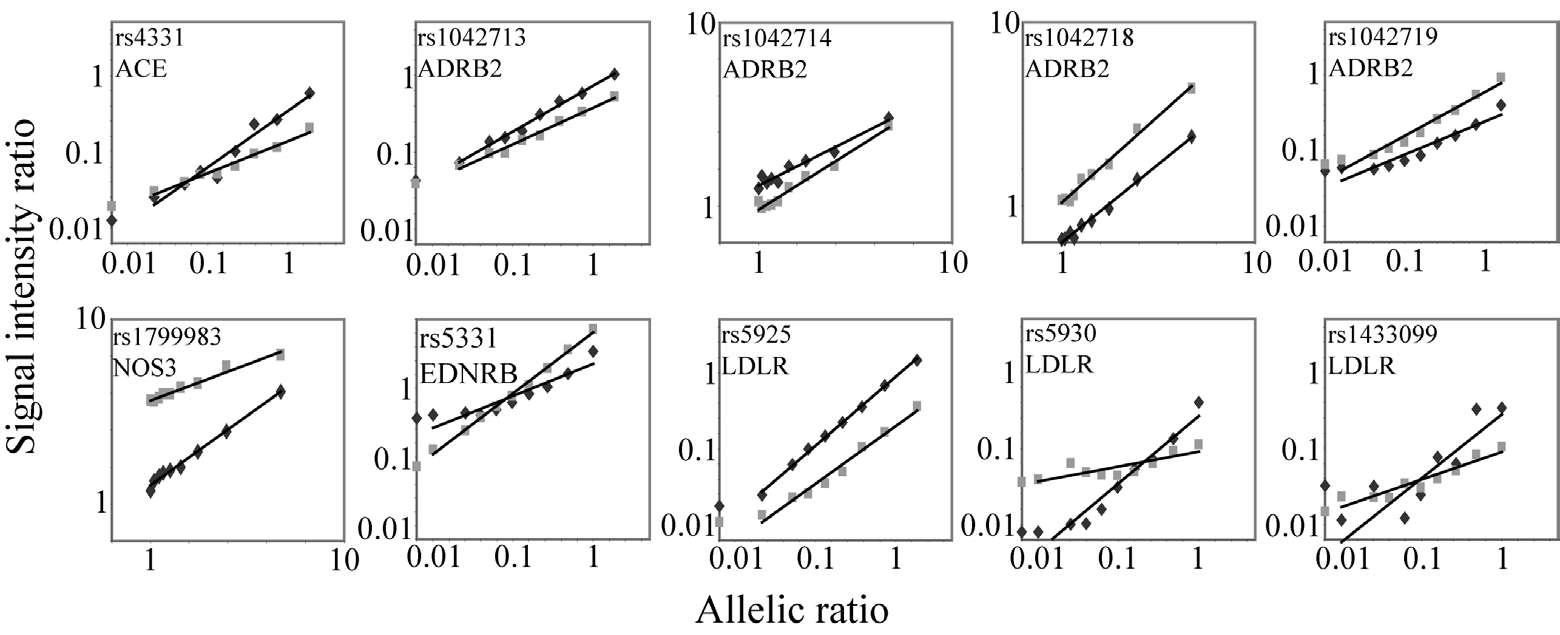
Regression lines displaying accuracy and sensitivity of quantitative genotyping of SNPs. The regression lines were obtained by analysing ten SNPs in a series of mixed samples with varying amounts of DNA from two individuals of different genotype. The signal intensity ratios from minisequencing using immobilised primers (Method I, black diamonds) and "tag-array" minisequencing (Method II, grey squares) are plotted as a function of the known allelic ratios in the mixed samples. The SNP names are given in the panels. The signal intensity ratios are mean values of five replicate reactions. The signal intensity ratios obtained in homozygous samples (allele ratios zero) are indicated as black diamonds and grey squares on the left vertical axis of each panel.

We analysed a dilution series with mixtures of DNA from two individuals with different genotypes for the panel of ten SNPs in both DNA polarities. The genotyping results from these mixtures of known amounts of the two SNP alleles are expressed as the signal ratio between the fluorescence signals corresponding to the two alleles of each SNP. The quantitative analysis of these ten SNPs is illustrated in Figure 1 by regression lines, in which the mean signal intensity ratios are plotted as a function of the known allelic ratios in the mixed samples. The coefficient of determination (R^2^), which describes how well the regression line fits the data points, was used to assess the accuracy of quantification of the SNP alleles by Methods I and II. As can be seen in Table 2, the R^2 ^values are close to one for most of the SNPs analysed, demonstrating little scatter of the data points around the regression line. For Method I, six of the ten SNPs analysed have R^2 ^values ≥ 0.95, while for Method II the R^2 ^values are ≥ 0.95 for eight of the SNPs. Thus, accurate quantification of SNP alleles is possible by both methods. The slopes of the regression lines vary between the ten SNPs as well as between the two methods (Figure 1). A regression line with a steep slope usually corresponds to a high R^2 ^value, as observed for the SNP rs5930 LDLR analysed by Method I and SNP rs5331 EDNRB analysed by Method II. A flat slope does not necessarily imply less accurate quantification, as exemplified by the SNP rs4331 ACE, where Method II yielded a flat slope with a higher R^2 ^value than Method I.

We also determined the sensitivity of the methods for detection of a minority allele. The detection limit was defined as the percentage of the minority allele in the mixed sample, for which the signal ratio differed from the signal ratio in the corresponding homozygous sample with a p-value < 0.05 in a two sample t-test. Depending on the genotype of the DNA samples used for the dilution series, determination of the lower limit of detection was possible for seven of the ten SNPs with allele ranges 0–50% or 0–100% in the mixed samples (Table 1). For the remaining three SNPs with the allele range 50–100%, the smallest percentage of an allele that could be distinguished from a heterozygous genotype was identified by the same approach. Using Method I, we were able to detect less than 5% of the minority allele for two SNPs (rs1042713 ADRB2 and rs5925 LDLR) and less than 9% for rs4331 ACE, rs1042719 ADRB2, rs5351 EDNRB and rs5930 LDLR (Table 2). Method II allowed more sensitive detection of minority alleles than Method I. Less than 2% was detectable for the SNPs rs1042713 ADRB2, rs1042719 ADRB2 and rs5351 EDNRB, and less than 9% was detectable for the SNPs rs4331 ACE, rs5925 LDLR, rs5930 LDLR and rs1433099 LDLR (Table 2). For the SNPs rs1042714 ADRB2, rs1042718 ADRB2 and rs1799983 NOS3, we were able to measure 4–14% deviations from the heterozygous genotype (Table 2). These results show that the amount of SNP alleles can be accurately determined on the DNA level by Methods I and II using reference samples with the two SNP alleles present in known ratios.

Next, the performance of the two methods in quantitative analysis on the RNA level was assessed. The ten SNPs were first genotyped in genomic DNA (gDNA) from the HUVEC and HAEC cells to identify those SNPs that were heterozygous in either or both cell lines. Three SNPs in the low density lipoprotein receptor gene (LDLR; rs5925, rs5930 and rs1433099) were heterozygous in the HAEC cell line, and one SNP in each of the genes encoding angiotensin I converting enzyme (ACE rs4331), β_2_-adrenergic receptor (ADRB2 rs1042719) and endothelin receptor type B (EDNRB rs5351) were heterozygous in the HUVEC cell line. These SNPs were genotyped in cDNA produced from total RNA extracted from the cells with the corresponding gDNA as reference samples using both methods. Table 3 presents the mean fluorescence signals with coefficients of variation (CV) obtained in five parallel reactions for the six SNPs in cDNA and gDNA from the HUVEC and HAEC cells. For the heterozygous SNPs the largest difference in the variability between parallel reactions was observed between SNPs, with the lowest CV values (3.6 – 8.6 %) for the rs1042719 ADRB2 SNP, and the highest CV values (13 – 41%) for the rs1433099 LDLR SNP. No systematic differences in the variability of parallel reactions were observed between Method I and Method II, or between cDNA and gDNA. Table 4 shows the differences in mean signal intensity ratios between the cDNA and gDNA assays for the six SNPs that were heterozygous in HUVEC or HAEC cells, respectively, together with the corresponding normalized cDNA/gDNA ratios. The SNPs in the ACE, ADRB2 and EDNRB genes displayed significant imbalanced expression in the HUVEC cells using both methods. For the SNP rs4331 ACE, the signal intensity ratio based on the raw data obtained by Methods I and II differed from each other, but despite this large difference, both methods yielded similar levels of allelic imbalance for this SNP after normalisation against the signal ratio in gDNA (Table 4). Only for one of the three LDLR SNPs (rs5930), the difference in fluorescence intensity ratios between cDNA and gDNA from HAEC cells reached statistical significance by both methods. Allelic imbalance of the LDLR gene was detected for the LDLR SNP rs5925 using Method II only.

To test that the results on imbalanced allelic expression detected by the multiplexed microarray based methods represents the true biological situation in the cells, we analysed the heterozygous SNPs in five replicate RNA samples prepared from HUVEC or HAEC harvested at different time points from different cell culture flasks. We also analysed the three LDLR SNPs in five replicate reverse transcription reactions from the same RNA sample prepared from HAEC cells. For this analysis we used our first generation solid-phase minisequencing assay for individual SNPs in a microtiter plate format. The concordant cDNA/gDNA ratios from these control experiments from independent cell and RNA samples presented in Table 5 show that the detected allelic imbalance was not caused by the procedures for RNA extraction or cDNA synthesis. Finally, we verified the results obtained by microarray-based minisequencing for three of the SNPs by real-time PCR with allele specific hybridization probes (TaqMan). Table 4 shows these results together with the corresponding results by solid-phase minisequencing in a microtiter plate format. Allelic imbalance was detected with statistical significance for the SNP rs1042719 ADRB2 and the SNP rs1433099 LDLR by both methods. Particularly for the SNP rs1042719 ADRB2, the cDNA/gDNA ratios obtained by the two reference methods were highly similar to the results from the microarray-based methods presented in Table 4, as well as with each other. As for the microarray-based Method II, the difference in signal ratios between cDNA and gDNA measured by the TaqMan assay for the SNP rs5925 LDLR did not reach statistical significance due to large variation between parallel assays. Analysis of the SNP rs1433099 LDLR by the reference methods confirms the imbalanced expression of the LDLR receptor alleles.

## Discussion

The purpose of our study was to evaluate microarray based minisequencing for multiplexed detection and quantification of imbalanced expression of SNP alleles, as a prelude to further large scale screening for allelic imbalance. We found no significant differences in the performance of our two "in house" methods, minisequencing with primers directly immobilised on the microarrays (Method I)[18] and the "tag-array" format, based on cyclic minisequencing followed by capture on microarrays using immobilised complementary "tag" probes (Method II) [23]. Both methods showed a linear relationship between SNP allele ratios and the signal intensity measured in the four-colour fluorescence minisequencing assay for all SNPs. With respect to accuracy assessed by coefficients of variation (CV) between five parallel assays both methods performed equally well, and the CV values between parallel assays were indistinguishable between genomic DNA and reverse transcribed cDNA samples. The sensitivity of detecting a SNP allele present as a minority in a sample was defined as the percentage for which the signal ratio differed from the signal ratio in the corresponding homozygous sample with a p-value < 0.05 in a two sample t-test. The sensitivity differed between SNPs, and range from 1% to 9%, with a trend to be slightly better using the "tag-array" system (Method II). In several cases the p-values were lower than 0.05 (Table 2), which indicates that in practice the sensitivity of detection would be lower than the stringent limit set here. The sensitivity of our multiplex microarray based minisequencing methods compares well with the sensitivity of other single nucleotide primer extension assays performed for individual SNPs in recent studies [4,25-27].

It is notable that the largest differences in accuracy and sensitivity were observed between SNPs. Some of the SNP-to-SNP differences are likely due to differences is the accuracy and efficiency of incorporation of the four different fluorescently labelled nucleotide analogues by the DNA polymerase [13,26] as well as to other sequence context dependent factors. The large variation between parallel assays for the SNP rs1433099 LDLR prevented detection of the allelic imbalance for the LDLR gene, while imbalance was detected by the SNP rs5930 LDLR using both methods. This result demonstrates that it is preferable to analyse more than a single SNP in each gene in systematic screening for allelic imbalance in gene expression. As more data from primer extension assays accumulate, it may be possible to improve the accuracy of the system by improving the SNP selection and assay design further with the aid of algorithms developed based on this data [28,29].

Comparison of the relative amounts of the alleles of six SNPs on the RNA (cDNA) level to heterozygote SNPs in genomic DNA revealed four SNPs with imbalanced expression of the two alleles. A three-fold increase in the expression of the T-allele for the SNP rs4331 ACE was the most pronounced difference observed. In our study, 1.4–1.5-fold differences in allelic expression levels were detectable. The sensitivity of detecting a minority allele in our system would allow the distinction between 10-fold reduction in the expression of an allele and monoallelic expression, for example as a result of imprinting. Owing to its potential for high throughput screening of large numbers of samples, we have also performed a preliminary evaluation of the commercial SNPstream genotyping system (GenomeLab, Beckman Coulter) that also utilises the "tag-array" primer extension strategy in a semi-automated 384-well microtiter plate format for detection of imbalanced allelic expression [30]. The same trend of imbalanced allelic expression was observed for each of the SNPs, which is encouraging for future studies of imbalanced allelic expression in a high throughput semi-automated way. Other studies that have used fluorescent single base primer extension assays report that 1.2 – fold to 1.5 – fold differences in allelic expression are detectable [2,4,5]. Primer extension methods based on direct measurement of fluorescent signals, including the microarray-based methods evaluated here, are likely to provide better accuracy and sensitivity for allele quantification than homogeneous primer extension based on fluorescence polarisation [31,32], in which the allele quantification relies on measurement of small differences between large polarization signals.

It is also reassuring for future large scale detection of imbalanced allelic expression that the accuracy of our methods seemed to be similar for cDNA and genomic DNA. Analysis of replicate RNA samples from different batches of both cell lines using a microtiter plate format of the minisequencing method evidenced for the biological authenticity of the allelic imbalance detected using minisequencing in the microarray format. The data obtained from independent cell samples also indicate an acceptable reproducibility of RNA extraction, RNA storage and cDNA synthesis. Another important factor besides sample to sample variation that may affect the accuracy of the relative allele quantification is the amount of mRNA subjected to the analysis. At a low copy number of mRNA, the stochastic distribution of the RNA templates may be a major source of variation [33]. The reason for the large variation between parallel assays for the LDLR receptor gene observed with all four methods used in our study may reflect a low expression level of the LDLR gene in the HAEC cells. Moreover, the amount of gene specific transcript in each RNA sample may vary which makes it difficult to perform balanced multiplex RT-PCRs to screen for allelic imbalances in several genes in one reaction.

A similar minisequencing strategy as the one used for determination of imbalanced expression between SNP alleles can also be used for determination of the relative expression levels of highly homologous genes [15] and for determination of alternatively spliced transcripts [34], a resolution that is beyond the capacity of traditional microarray based RNA expression profiling.

## Conclusions

Here we demonstrated the applicability of two formats of microarray based minisequencing for detecting imbalanced expression of SNP alleles. The accuracy and sensitivity of both systems allow detection of 1.4- to 10-fold differences in the expression levels of the two alleles of heterozygous SNPs. The microarray-based minisequencing systems utilise widely available reagents and equipment, and can thus easily be established "in-house". Moreover, the system is flexible with respect to number of SNPs and samples to be analyzed. Systematic quantitative screening of genetic diversity on the RNA level in multiple individuals and tissues will be a future approach in the elucidation of the molecular mechanisms that regulate gene expression.

## Methods

### DNA and RNA samples

DNA samples from 30 volunteer donors were genotyped by Methods I and II to identify individuals of different genotypes for the panel of ten SNPs analysed. The SNPs are described in the section "SNPs and primers" below. DNA (10 ng/μl) from one individual was serially diluted 2:1 into DNA (10 ng/μl) from a second individual, to yield a series of DNA samples with different ratios between the SNP alleles. These mixed DNA samples were used for construction of quantification standard curves. Depending on the genotype of each SNP in the two individuals whose DNA was mixed, dilution series of samples with different allelic ranges were obtained for the ten SNPs, as specified in Table 1.

Human Umbilical Vein Endothelial Cells (HUVEC) and Human Aortic Endothelial Cells (HAEC) (Cascade Biologics, Inc., Portland, OR, USA) were grown in Medium 200 with Low Serum Growth Supplement (LSGS Kit, Cascade Biologics, Inc., Portland, OR, USA) at 37°C in a humidified atmosphere of 5% CO_2_. Cells from the cultures were harvested at 80% confluence according to the manufacturer's instructions. Total RNA was isolated from the cells using the TRIZOL^®^Reagent (GIBCO BRL, Paisley, Scotland) and the RNA samples were stored at -70°C until use. High quality RNA with A_260_/A_280 _ratio over 1.9 and intact ribosomal 28S and 18S RNA were used for cDNA synthesis. The RNA samples were treated with 1 U RQ1 RNase-free DNase (Promega, Madison, WI, USA) per μg RNA. Two to 2.5 μg total RNA was subjected to first strand cDNA synthesis using SuperScript™ II (RNase H^- ^Reverse Transcriptase, Invitrogen, Carlsbad, CA, USA) reagents in a 20 μl volume. DNA was extracted from the cells using GenElute™ Mammalian Genomic DNA Kit (Sigma, St Louis, MO, USA) and stored at -20°C until use.

### PCR

The fragments comprising the SNPs were PCR-amplified in individual reactions using 10–15 ng genomic DNA or one tenth of the cDNA products, 0.2 mM dNTPs, 1U AmpliTaq ^® ^Gold DNA polymerase (Applied Biosystems, Foster City, CA, USA), 1.5 mM MgCl_2_, and 0.2–0.3 μM of primers in 50 μl of 10 mM Tris-HCl pH 8.3 and 50 mM KCl. The PCR conditions were initial activation of the enzyme at 95°C for 10 min followed by 35 cycles of 95°C for 1 min, 56°C for 1 min and 72°C for 1 min and a final extension at 72°C for 7 min in a Thermal Cycler PTC225 (MJ Research, Watertown, MA, USA). The amplified fragments were combined and concentrated to 60 μl using Microcon ^® ^YM-30 Centrifugal Filter Devices (Millipore Corporation, Bedford, MA, USA).

### SNPs and primers

Ten SNPs located in coding regions of genes known to be expressed in HUVEC and HAEC cells were analysed. Information on the SNPs, including dbSNP [35] ID number and nucleotide variation is given [see Additional file 1] together with the sequences of the minisequencing primers. The primers for PCR and minisequencing were designed using the Oligo Primer Analysis software v6.65 (Molecular Biology Insights Inc., Cascade, CO, USA).

### Preparation of microarrays

The minisequencing primers or the complementary tag-oligonucleotides were covalently immobilised on CodeLink™ Activated Slides (Amersham Biosciences, Uppsala, Sweden) by the mediation of a NH_2_-group in their 5'- or 3'-end, respectively. The oligonucleotides were applied in duplicates to the slides at a concentration of 25 μM in 150 mM sodium phosphate pH 8.5 using a ProSys 5510A instrument (Cartesian Technologies Inc, Irvine. CA, USA) equipped with one Stealth Micro Spotting pin (SMP3B, TeleChem International Inc., Sunnyvale, CA, USA) to minimise the variation between spots in different "subarrays". The oligonucleotides were spotted in an "array-of-arrays" configuration that facilitates analysis of 80 individual samples in parallel on each microscope slide [24]. In each "subarray" a fluorophore-labelled oligonucleotide was included as a control for the immobilisation process. A reference oligonucleotide, complementary to a synthetic template included in the minisequencing reaction mixtures to monitor the difference in incorporation efficiency of the four nucleotides by the DNA polymerase, was also included in each "subarray". Finally, an oligonucleotide designed not to hybridise to any of the oligonucleotides present in the reaction mixture was included in each "sub-array" to be used for background corrections. After printing, the slides were incubated in a humid chamber for at least 24 hours, followed by treatment with ethanolamine according to the manufacturer's instruction. The slides were then stored desiccated in the dark until use.

### Minisequencing using immobilised primers (Method I)

Aliquots of 7.5 μl of the concentrated PCR products were analysed in five parallel "subarrays" for each sample, essentially as described previously [18]. The PCR products were allowed to anneal to the immobilised oligonucleotides. After washing, the extension reactions were performed with 0.75 U of Thermo Sequenase™ DNA polymerase (Amersham Biosciences, Uppsala, Sweden) and 0.35 μM Texas Red-ddATP, Tamra-ddCTP, R110-ddGTP and Cy5-ddUTP (Perkin Elmer Life Sciences, Boston, MA, USA) in Thermo Sequenase™ reaction buffer in a total volume of 15 μl, followed by washing of the slide.

### Minisequencing using "tag-arrays" (Method II)

Five parallel reactions with a 4.5 μl aliquot of the concentrated PCR products were analysed for each sample, as described in detail in [23]. Excess of PCR primers and dNTPs were removed by treatment with 5 U of exonuclease I and 1 U of shrimp alkaline phosphatase (USB Corporation, Cleveland, OH, USA). The cyclic minisequencing reactions were performed in the presence of the 20 tagged primers at 10 nM concentration, 0.1 μM Texas Red-ddATP, Tamra-ddCTP and R110-ddGTP, 0.2 μM Cy5-ddUTP (Perkin Elmer Life Sciences, Boston, MA, USA) and 1 U of Thermo Sequenase™ DNA polymerase (Amersham Biosciences, Uppsala, Sweden) for 55 cycles of 95°C and 55°C for 20 s each. The extension products were allowed to anneal to the immobilised complementary tag oligonucleotides at 42°C for 2.5 hours followed by washing of the slide.

### Solid-phase minisequencing in a microtiter plate format

PCR was run with one of the primers biotinylated. The biotinylated PCR products were immobilised in a microtiter plate coated with streptavidin (Combiplate 8, Labsystems, Helsinki, Finland) and the unbiotinylated strand was removed with alkali treatment [9,15]. The minisequencing mixture, containing the appropriate tritium labelled dNTP (Amersham Biosciences, Uppsala, Sweden), AmpliTaq ^® ^DNA polymerase (Applied Biosystems, Foster City, CA, USA) and the minisequencing primer was added. The extension reaction was allowed to proceed for 10 min at 50°C. The extended primers were released with alkali and the amount of incorporated tritium labelled nucleotide was measured.

### Hybridisation with allele-specific TaqMan probes

Primers and probes for the TaqMan assays were designed by Applied Biosystems as Assay-by-Design (rs1042719 ADRB2 and rs5925 LDLR) or Assay-on-Demand (rs1433099 LDLR) service. The probes for the two alleles were labelled with the reporter dyes FAM and VIC respectively. The sequences of the primers and probes for the SNPs rs5925 LDLR and rs 1042719 ADRB2 are found in [Additional file 1]. The primer and probe sequences for the SNP rs1433099 LDLR were not made available to us by ABI since this SNP is included in their Assay-on-Demand program.

Real time quantitative PCR was run in 25 μl TaqMan Universal PCR Master Mix (Applied Biosystems) with 200 nM of both labelled TaqMan probes, 900 nM PCR-primers and 10 ng genomic DNA or one tenth of the cDNA products. The PCR conditions were initial activation of the enzyme at 95°C for 10 min followed by 60 cycles of 95°C for 15 sec and 60°C for 1 min in a ABI7000 instrument (Applied Biosystems, Foster City, CA, USA).

The signal intensity ratios were calculated based on normalised ΔRn fluorescence values obtained from the assay during the exponential phase of PCR. The ΔRn values were retrieved from cycle 38 for the SNP rs1042719 ADRB2, cycle 42 for the SNP rs5925 LDLR and cycle 43 for the SNP rs1433099 LDLR. Imbalanced expression of the SNP alleles was determined by a t-test as described below.

### Signal detection and data analysis

In Methods I and II fluorescence was measured using a ScanArray ^® ^Express instrument (Perkin Elmer Life Sciences, Boston, MA, USA) with the excitation lasers Blue Argon 488 nm, Green HeNe 543.8 nm, Yellow HeNe 594 nm and Red HeNe 632.8 nm with the laser power set to 80% and the photomultiplier tube gain adjusted to obtain equal signal intensities from reaction control spots for all four spectra. The fluorescence signals were extracted using the QuantArray ^® ^analysis 3.1 software (Perkin Elmer Life Sciences, Boston, MA, USA). The mean of the fluorescence signals for the duplicate spots was corrected for the average background in each "sub-array" separately. The data was handled and interpreted using the Microsoft ^® ^Excel program.

The genotype for each individual SNP was assigned by calculating a ratio between the fluorescence signals for the two alleles. Coefficients of determination (R^2^) were assigned by linear regression analysis of the relationship between the signal intensity ratios determined from the minisequencing assay and the known allelic ratios in the mixed samples for the quantification standard curves. Two-sample t-tests with two-tailed significance levels assuming unequal variance were performed to determine the lowest level of detection of a specific allele for the quantification standard curves and to evaluate the imbalanced expression of the two alleles of the SNPs in the cell lines.

## Authors' contributions

UL participated in the design of the study and in RNA and DNA extraction, and performed all the laboratory work involving "in-house" minisequencing methods, performed the statistical calculations and drafted the manuscript. MF cultured the cells, performed RNA and DNA extraction, performed the assays with the reference method, and provided input to the manuscript. AD performed the assays using the SNPstream system. A-CS conceived the study, participated in its design, coordination and in preparation of the manuscript. All authors read and approved the final manuscript.

## Supplementary Material

Additional File 1 Additional file1 is a pdf-file with information on the SNPs, including dbSNP ID number, nucleotide variation and the sequences of the primers and probes used in the microarray based minisequencing and TaqMan assays respectively. Click here for file

## Figures and Tables

**Table 1:** SNP genotypes of the DNA samples used for preparing the dilution series for the quantification curves.

SNP^a^	Sample 1	Sample 2	Allele range^b^
rs4331 ACE	TT	CC	0–100% T
rs1042713 ADRB2	AA	GG	0–100% A
rs1042714 ADRB2	CC	CG	50–100% C
rs1042718 ADRB2	CC	CA	50–100% C
rs1042719 ADRB2	CG	GG	0–50% C
rs1799983 NOS3	CC	CA	50–100% C
rs5351 EDNRB	GA	AA	0–50% G
rs5925 LDLR	TT	CC	0–100% T
rs5930 LDLR	TC	CC	0–50% T
rs1433099 LDLR	TC	CC	0–50% T

^a ^SNP name consisting of dbSNP ID number and gene name acronym.

^b ^The percentages of the allele from Sample 1 in the dilution series with the 0–100% allele range were: 0%; 2.1%; 4.9%; 7.5%; 11.6%; 17.9%; 27.5%; 42.3%; 65%; 100%. The corresponding percentages in the 0–50% allele range were 0%; 1.0%; 2.5%; 3.8%; 5.8%; 8.9%; 13.7%; 21.1%; 32.5%; 50%, and in the 50–100% allele range they were 50%; 51.3%; 52.5%; 53.8%; 55.8%; 58.9%; 63.7%; 71.1%; 82.%; 100%.

**Table 2:** Results for the regression lines describing the accuracy and limit of detection for the methods when analysing mixed samples of a dilution series.

SNP^a^	Method^b^	R^2c^	Detection sensitivity (%)^d ^p < 0.05	p-value^e^
rs4331 ACE	I	0.95	7.5	0.0076
	II	0.97	4.9	0.0107
rs1042713 ADRB2	I	0.99	4.9	0.00051
	II	0.98	2.1	0.000077
rs1042714 ADRB2	I	0.96	14 *	0.042
	II	0.98	8.9 *	0.047
rs1042718 ADRB2	I	0.99	14 *	0.020
	II	0.99	5.8 *	0.0011
rs1042719 ADRB2	I	0.90	5.8	0.0072
	II	0.96	1.0	0.013
rs1799983 NOS3	I	0.99	8.9 *	0.0015
	II	0.97	3.8 *	0.023
rs5351 EDNRB	I	0.87	5.8	0.016
	II	1.0	1.0	0.000016
rs5925 LDLR	I	1.0	4.9	0.014
	II	0.98	4.9	0.018
rs5930 LDLR	I	0.94	8.9	0.00030
	II	0.63	33	0.011
rs1433099 LDLR	I	0.75	ND	ND
	II	0.88	5.8	0.017

^a ^dbSNP ID number and gene name acronym.

^b ^Minisequencing using (I) immobilised primers; (II) cyclic primer extension and "tag-arrays".

^c ^Coefficient of determination describing the fit between the regression lines in Figure 1, and the data points.

^d ^Level at which the minority SNP allele can be detected. The percentages correspond to the mixture with a signal intensity ratio significantly different (p < 0.05) from the signal intensity ratio of the corresponding homozygous or heterozygous (*) sample. ND: Not possible to determine due to scatter of the data points.

^e ^p-value for difference between signal ratios at the detection level and at 0% of the minority allele.

**Table 3:** Fluorescence signals obtained by genotyping six SNPs in RNA (cDNA) and genomic DNA from the HUVEC and HAEC cells using the two minisequencing methods.

		Fluorescence signal (CV %)^b^								Fluorescence signal ratio^c^	
		cDNA				gDNA				cDNA	gDNA
SNP^a^	Method	Allele 1		Allele 2		Allele 1		Allele 2			
rs4331	I	17975	(31%)	4428	(20%)	17568	(12%)	10291	(10%)	4.1	1.7
ACE	II	20447	(37%)	21796	(11%)	8928	(35%)	26913	(18%)	0.94	0.33
rs1042719	I	14368	(12%)	28538	(25%)	21179	(11%)	22192	(12%)	0.50	0.95
ADRB2	II	11475	(3.6%)	34346	(5.4%)	18495	(7.4%)	39569	(8.6%)	0.33	0.47
rs5351	I	11026	(10%)	1743	(15%)	7344	(16%)	2077	(17%)	6.3	3.5
EDNRB	II	65257	(0%)	7484	(14%)	64360	(3.3%)	11552	(20%)	8.7	5.6
rs5925	I	13984	(25%)	16040	(5.7%)	9586	(32%)	9329	(20%)	0.87	1.0
LDLR	II	3504	(27%)	9582	(14%)	1951	(10%)	7113	(8.2%)	0.37	0.27
rs5930	I	5680	(13%)	5028	(13%)	7207	(22%)	9244	(17%)	1.1	0.78
LDLR	II	5410	(31%)	9965	(15%)	3261	(27%)	11183	(9.0%)	0.54	0.29
rs1433099	I	8806	(20%)	4594	(10%)	7307	(32%)	3646	(23%)	2.0	2.0
LDLR	II	2550	(41%)	4507	(16%)	1727	(36%)	3743	(13%)	0.57	0.46

^a ^rs4331 ACE, rs1042719 ADRB2 and rs5351 EDNRB were analysed in HUVEC and rs5925 DLR, rs5930 LDLR and rs1433099 LDLR were analysed in HAEC.

^b ^Mean values and coefficient of variation (CV) of five parallel minisequencing assays from the same PCR product.

^c ^The homozygous signal intensity ratios were 0.0027 for rs4331 ACE, 0.0094 for rs1042719 ADRB2, and 0.10 for rs5351 EDNRB in gDNA from HAEC, and 0.0069 for rs5925 LDLR, 0.0056 for rs5930 LDLR, and 0.014 for rs1433099 LDLR in gDNA from HUVEC.

**Table 4:** Fluorescence signal intensity ratios obtained by genotyping six SNPs in RNA (cDNA) and genomic DNA from the HUVEC and HAEC cells using the two microarray-based minisequencing methods and two reference methods.

	Fluorescence signal ratio			Difference in cDNA and gDNA ratios (p-value)	cDNA/gDNA^c^
SNP^a^	Method^b^	cDNA	gDNA		
rs4331 ACE	I	4.1	1.7	0.00095	2.4 (1.9–2.8)
	II	0.94	0.33	0.0060	2.8 (1.8–4.0)
rs1042719 ADRB2	I	0.50	0.95	0.000025	0.54 (0.47–0.69)
	II	0.33	0.47	0.0070	0.71 (0.67–0.75)
	TM	0.086	0.19	<0.0001	0.47 (0.31–0.56)
	MS	0.48	0.99	<0.0001	0.48 (0.34–0.62)
rs5351 EDNRB	I	6.3	3.5	0.00014	1.8 (1.6–2.0)
	II	8.7	5.6	0.0030	1.6 (1.4–1.8)
rs5925 LDLR	I	0.87	1.0	0.33	0.85 (0.63–1.1)
	II	0.37	0.27	0.029	1.4 (1.1–1.5)
	TM	3.3	1.4	0.15	2.4 (1.4–5.6)
	MS	0.36	0.28	0.027	1.3 (0.98–1.5)
rs5930 LDLR	I	1.1	0.78	0.035	1.5 (1.1–1.9)
	II	0.54	0.29	0.0030	1.8 (1.5–2.3)
rs1433099 LDLR	I	2.0	2.0	0.66	0.96 (0.84–1.1)
	II	0.57	0.46	0.33	1.2 (0.65–1.5)
	TM	1.0	0.49	<0.0001	2.0 (1.9–2.1)
	MS	0.47	0.32	0.060	1.5 (1.0–2.0)

^a ^rs4331 ACE, rs1042719 ADRB2 and rs5351 EDNRB were analysed in HUVEC and rs5925 LDLR, rs5930 LDLR and rs1433099 LDLR were analysed in HAEC.

^b ^Minisequencing using (I) immobilised primers; (II) cyclic primer extension and "tag-arrays", TaqMan (TM) and solid-phase minisequencing (MS). TM-assays were not possible to design using the Assay-by-Design and Assay-on-Demand service at ABI for the remaining three SNPs, MS was performed for the SNPs with working TM-assays.

^c ^Mean and range is given for five parallel reactions.

**Table 5:** Test of allelic imbalance in replicate RNA-preparations and cDNA samples.

	cDNA/gDNA ratios in replicate RNA preparations ^a^		

	rs4331 ACE	rs1042719 ADRB2	rs5351 EDNRB
	
HUVEC	2.8 (1.4 – 4.0)	0.48 (0.34 – 0.62)	1.2 (1.1 – 1.4)

	rs5925 LDLR	rs5930 LDLR	rs1433099 LDLR
	
HAEC	1.3 (0.98 – 1.5)	1.6 (1.0 – 2.3)	1.5 (1.0 – 2.0)

	cDNA/gDNA ratios in replicate cDNA samples ^b^		

	rs5925 LDLR	rs5930 LDLR	rs1433099 LDLR
	
HAEC	1.5 (1.3 – 1.7)	1.9 (1.7 – 2.4)	1.1 (0.87 – 1.4)

Average cDNA/gDNA ratios from five replicate experiments with range of variation in parantheses.

^a ^cDNA synthesised from RNA extracted from five different cell batches analysed in duplicate using solid-phase minisequencing assays in a microtiter plate format.

^b ^Five replicate cDNA samples were reverse transcribed from the same RNA preparation and analysed in duplicate assays.

## References

[B1] Sachidanandam R, Weissman D, Schmidt SC, Kakol JM, Stein LD, Marth G, Sherry S, Mullikin JC, Mortimore BJ, Willey DL, Hunt SE, Cole CG, Coggill PC, Rice CM, Ning Z, Rogers J, Bentley DR, Kwok PY, Mardis ER, Yeh RT, Schultz B, Cook L, Davenport R, Dante M, Fulton L, Hillier L, Waterston RH, McPherson JD, Gilman B, Schaffner S, Van Etten WJ, Reich D, Higgins J, Daly MJ, Blumenstiel B, Baldwin J, Stange-Thomann N, Zody MC, Linton L, Lander ES, Attshuler D (2001). A map of human genome sequence variation containing 1.42 million single nucleotide polymorphisms. Nature.

[B2] Yan H, Yuan W, Velculescu VE, Vogelstein B, Kinzler KW (2002). Allelic variation in human gene expression. Science.

[B3] Lo HS, Wang Z, Hu Y, Yang HH, Gere S, Buetow KH, Lee MP (2003). Allelic variation in gene expression is common in the human genome. Genome Res.

[B4] Pastinen T, Sladek R, Gurd S, Sammak A, Ge B, Lepage P, Lavergne K, Villeneuve A, Gaudin T, Brandstrom H, Beck A, Verner A, Kingsley J, Harmsen E, Labuda D, Morgan K, Vohl MC, Naumova AK, Sinnett D, Hudson TJ (2004). A survey of genetic and epigenetic variation affecting human gene expression. Physiol Genomics.

[B5] Bray NJ, Buckland PR, Owen MJ, O'Donovan MC (2003). Cis-acting variation in the expression of a high proportion of genes in human brain. Hum Genet.

[B6] Yan H, Zhou W (2004). Allelic variations in gene expression. Curr Opin Oncol.

[B7] Hudson TJ (2003). Wanted: regulatory SNPs. Nat Genet.

[B8] Syvanen AC (2001). Accessing genetic variation: genotyping single nucleotide polymorphisms. Nat Rev Genet.

[B9] Syvanen AC, Sajantila A, Lukka M (1993). Identification of individuals by analysis of biallelic DNA markers, using PCR and solid-phase minisequencing. Am J Hum Genet.

[B10] Ross P, Hall L, Haff LA (2000). Quantitative approach to single-nucleotide polymorphism analysis using MALDI-TOF mass spectrometry. Biotechniques.

[B11] Buetow KH, Edmonson M, MacDonald R, Clifford R, Yip P, Kelley J, Little DP, Strausberg R, Koester H, Cantor CR, Braun A (2001). High-throughput development and characterization of a genomewide collection of gene-based single nucleotide polymorphism markers by chip-based matrix-assisted laser desorption/ionization time-of-flight mass spectrometry. Proc Natl Acad Sci U S A.

[B12] Werner M, Sych M, Herbon N, Illig T, Konig IR, Wjst M (2002). Large-scale determination of SNP allele frequencies in DNA pools using MALDI-TOF mass spectrometry. Hum Mutat.

[B13] Lindroos K, Sigurdsson S, Johansson K, Ronnblom L, Syvanen AC (2002). Multiplex SNP genotyping in pooled DNA samples by a four-colour microarray system. Nucleic Acids Res.

[B14] Singer-Sam J, LeBon JM, Dai A, Riggs AD (1992). A sensitive, quantitative assay for measurement of allele-specific transcripts differing by a single nucleotide. PCR Methods Appl.

[B15] Karttunen L, Lonnqvist L, Godfrey M, Peltonen L, Syvanen AC (1996). An accurate method for comparing transcript levels of two alleles or highly homologous genes: application to fibrillin transcripts in Marfan patients' fibroblasts. Genome Res.

[B16] Matyas G, Giunta C, Steinmann B, Hossle JP, Hellwig R (2002). Quantification of single nucleotide polymorphisms: A novel method that combines primer extension assay and capillary electrophoresis. Hum Mutat.

[B17] Pastinen T, Kurg A, Metspalu A, Peltonen L, Syvanen AC (1997). Minisequencing: a specific tool for DNA analysis and diagnostics on oligonucleotide arrays. Genome Res.

[B18] Liljedahl U, Karlsson J, Melhus H, Kurland L, Lindersson M, Kahan T, Nystrom F, Lind L, Syvanen AC (2003). A microarray minisequencing system for pharmacogenetic profiling of antihypertensive drug response. Pharmacogenetics.

[B19] Chen J, Iannone MA, Li MS, Taylor JD, Rivers P, Nelsen AJ, Slentz-Kesler KA, Roses A, Weiner MP (2000). A microsphere-based assay for multiplexed single nucleotide polymorphism analysis using single base chain extension. Genome Res.

[B20] Fan JB, Chen X, Halushka MK, Berno A, Huang X, Ryder T, Lipshutz RJ, Lockhart DJ, Chakravarti A (2000). Parallel genotyping of human SNPs using generic high-density oligonucleotide tag arrays. Genome Res.

[B21] Lindroos K, Liljedahl U, Raitio M, Syvanen AC (2001). Minisequencing on oligonucleotide microarrays: comparison of immobilisation chemistries. Nucleic Acids Res.

[B22] Lindpaintner K (2002). The impact of pharmacogenetics and pharmacogenomics on drug discovery. Nat Rev Drug Discov.

[B23] Lovmar L, Fredriksson M, Liljedahl U, Sigurdsson S, Syvanen AC (2003). Quantitative evaluation by minisequencing and microarrays reveals accurate multiplexed SNP genotyping of whole genome amplified DNA. Nucleic Acids Res.

[B24] Pastinen T, Raitio M, Lindroos K, Tainola P, Peltonen L, Syvanen AC (2000). A system for specific, high-throughput genotyping by allele-specific primer extension on microarrays. Genome Res.

[B25] Hochberg EP, Miklos DB, Neuberg D, Eichner DA, McLaughlin SF, Mattes-Ritz A, Alyea EP, Antin JH, Soiffer RJ, Ritz J (2003). A novel rapid single nucleotide polymorphism (SNP)-based method for assessment of hematopoietic chimerism after allogeneic stem cell transplantation. Blood.

[B26] Norton N, Williams NM, Williams HJ, Spurlock G, Kirov G, Morris DW, Hoogendoorn B, Owen MJ, O'Donovan MC (2002). Universal, robust, highly quantitative SNP allele frequency measurement in DNA pools. Hum Genet.

[B27] Mohlke KL, Erdos MR, Scott LJ, Fingerlin TE, Jackson AU, Silander K, Hollstein P, Boehnke M, Collins FS (2002). High-throughput screening for evidence of association by using mass spectrometry genotyping on DNA pools. Proc Natl Acad Sci U S A.

[B28] Yuryev A, Huang J, Pohl M, Patch R, Watson F, Bell P, Donaldson M, Phillips MS, Boyce-Jacino MT (2002). Predicting the success of primer extension genotyping assays using statistical modeling. Nucleic Acids Res.

[B29] Kaderali L, Deshpande A, Nolan JP, White PS (2003). Primer-design for multiplexed genotyping. Nucleic Acids Res.

[B30] Bell PA, Chaturvedi S, Gelfand CA, Huang CY, Kochersperger M, Kopla R, Modica F, Pohl M, Varde S, Zhao R, Zhao X, Boyce-Jacino MT, Yassen A (2002). SNPstream UHT: ultra-high throughput SNP genotyping for pharmacogenomics and drug discovery. Biotechniques.

[B31] Chen X, Levine L, Kwok PY (1999). Fluorescence polarization in homogeneous nucleic acid analysis. Genome Res.

[B32] Kwok PY (2002). SNP genotyping with fluorescence polarization detection. Hum Mutat.

[B33] Stenman J, Orpana A (2001). Accuracy in amplification. Nat Biotechnol.

[B34] Zhu J, Shendure J, Mitra RD, Church GM (2003). Single molecule profiling of alternative pre-mRNA splicing. Science.

[B35] The NCBI SNP database. http://www.ncbi.nlm.nih.gov/SNP/.

