# Supplementary material for: Detecting imbalanced expression of SNP alleles by minisequencing on microarrays
Source: BMC Biotechnol. 2004 Oct 22;4:24. doi: 10.1186/1472-6750-4-24 (PMC529269; doi:10.1186/1472-6750-4-24)
Supplement: Additional File 1 — Additional file1 is a pdf-file with information on the SNPs, including dbSNP ID number, nucleotide variation and the sequences of the primers and probes used in the microarray based minisequencing and TaqMan assays respectively. [file 1472-6750-4-24-S1.pdf]

Additional file 1. SNP information and sequences of primers and probes.

Part A. SNP information and sequences of the minisequencing primers.

SNP[a]	Variation[b]	Minisequencing primer Method I[c]	Minisequencing primer Method II[c]
rs4331 ACE		CAGGACCTAGAACGGGCAGC	gtatcatgttatagggctgcCAGGACCTAGAACGGGCAGC
	T/C	TCCAGCTCCTGGGCAGGCAG	gactctgtcgatagctgtgtTCCAGCTCCTGGGCAGGCAG
rs1042713 ADRB2	A/G	CCTTCTTGCTGGCACCCAAT	ctctcttagcatagtgtcggCCTTCTTGCTGGCACCCAAT
		GTGGTCCGGCGCATGGCTTC	gcatcctatcttacgtgtcaGTGGTCCGGCGCATGGCTTC
rs1042714 ADRB2	C/G	CGGACCACGACGTCACGCAG	catgtatagacagtgcgcgtCGGACCACGACGTCACGCAG
		CACCCACACCTCGTCCCTTT	atcatataggatgcggcgatCACCCACACCTCGTCCCTTT
rs1042718 ADRB2	C/A	CCATTCAGATGCACTGGTAC	ctcatatagcgttacggtcgCCATTCAGATGCACTGGTAC
		GGCTTCCTGGTGGGTGGCCC	ccgatcaatgagtctagtgtGGCTTCCTGGTGGGTGGCCC
rs1042719 ADRB2	C/G	TCTTCTTTGAAGGCCTATGG	ctcattagtatcatcgtcggGTCTTCTTTGAAGGCCTATGG
		CCGTTGCTGGAGTAGCCATT	gcgttattcagatcgagtgaCCGTTGCTGGAGTAGCCATT
rs5351 EDNRB	G/A	ACAGCAAAAGATTGGTGGCT	agtctgatcccatcgcgtatACAGCAAAAGATTGGTGGCT
		AAGCAGAAATAGAAACTGAA	agcttagcgacaatgcctttGCAAGCAGAAATAGAAACTGAA

rs1799983 NOS3		CTGCTGCAGGCCCCAGATGA	cgtgccgctcgtgatagaatCTGCTGCAGGCCCCAGATGA
	C/A	AGAAGGAAGAGTTCTGGGGG	gctagatcgtgcgttatatgAGAAGGAAGAGTTCTGGGGG
rs5925 LDLR	T/C	CTGTCCCCAGAGGATATGGT	atactggctacacgtccataCTGTCCCCAGAGGATATGGT
		TGGGTGAGGTTGTGGAAGAG	gaagtctgtcctcctctataTGGGTGAGGTTGTGGAAGAG
rs5930 LDLR		TATGACACCGTCATCAGCAG	aggtcggctgcacgctaaatTATGACACCGTCATCAGCAG
	T/C	CCGTCGGGGGCCTGGATGTC	aggtcgcatggacgaacatcCCGTCGGGGGCCTGGATGTC
rs1433099 LDLR	T/C	AGAGGCAGAGCCTGAGTCAC	gaggtcagttcacgaagctcAGAGGCAGAGCCTGAGTCAC
		CTTAATAAATATTAAGGGTGACC	gaggtcgcttcactactcttCTTAATAAATATTAAGGGTGACC

[a] dbSNP ID number (http://www.ncbi.nlm.gov/SNP). Gene acronym according to HUGO Gene Nomenclature Committee (http://www.gene.ucl.ac.uk/nomenclature/).

[b] Nucleotide variations in the strand for which the results are presented. Coding strand for all SNPs except rs4331 ACE, rs1799983 ECNOS and rs5930 LDLR.

[c] For Method I each primer has a 5’tail of 15 T-residues. For Method II the twenty 5’ nucleotides (shown in lower case) are a Tag sequence from the Affymetrix GenFlex™ Tag collection (Affymetrix, Santa Clara, CA, USA) For each SNP the primer sequence corresponding to the coding DNA strand is given above that for the non-coding strand.

Part B. Sequences of the primers and probes for the TaqMan assays.

SNP [a]	PCR-primer [b]	TaqMan probe [b]
rs1042719 ADRB2	GCCTTCCAGGAGCTTCTGT	CCTATGGCAATGGCT
	ACTCTGCTCCCCTGTGTTG	CCTATGGGAATGGCT
rs5925 LDLR	CCTCACAGGTTCCGATGTCAA	TTGTGGAAGAGGACCATA
	CTGACCCACCCTTACCTCTTG	TGTGGAAGAGAACCATA
rs1433099 LDLR	Assay-on-Demand	Assay-on-Demand

[a] dbSNP ID number (http://www.ncbi.nlm.gov/SNP). Gene acronym according to HUGO Gene Nomenclature Committee (http://www.gene.ucl.ac.uk/nomenclature/).

[b] For each SNP the primer sequence corresponding to the coding DNA strand is given above that for the non-coding strand
